# Identification of Two Porcine Reproductive and Respiratory Syndrome Virus Variants Sharing High Genomic Homology but with Distinct Virulence

**DOI:** 10.3390/v11090875

**Published:** 2019-09-18

**Authors:** Nanhua Chen, Mengxue Ye, Yucheng Huang, Shuai Li, Yanzhao Xiao, Xinshuai Li, Shubin Li, Xiangdong Li, Xiuling Yu, Kegong Tian, Jianzhong Zhu

**Affiliations:** 1 College of Veterinary Medicine, Yangzhou University, Yangzhou, Jiangsu 225009, China; 2 National Research Center for Veterinary Medicine, High-Tech District, Luoyang, Henan 471003, China

**Keywords:** Porcine reproductive and respiratory syndrome virus, high homology, deletion, distinct virulence, potential virulence-associated amino acids

## Abstract

Porcine reproductive and respiratory syndrome virus (PRRSV) causes huge economic loss to the global swine industry. Even though several control strategies have been applied, PRRS is still not effectively controlled due to the continuous emergence of new variants and limited cross-protection by current vaccines. During the routine epidemiological investigation in 2017, two PRRSV variants were identified from a severe abortion farm and a clinically healthy farm, respectively. The viruses were isolated and denominated as XJ17-5 and JSTZ1712-12. Genomic sequencing indicated that their genomes are both 14,960 bp in length sharing 99.45% nucleotide identity. Sequence alignments identified a discontinuous 30-amino-acid deletion and a continuous 120-amino-acid deletion in *nsp2* of both isolates. Genome-based phylogenetic analysis confirmed that XJ17-5 and JSTZ1712-12 belong to the HP-PRRSV subtype but form a new branch with other isolates containing the same 150-amino-acid deletion in *nsp2*. Pathogenic analysis showed that XJ17-5 is highly virulent causing 60% mortality, while JSTZ1712-12 is avirulent for piglets. Furthermore, fragment comparisons identified 34-amino-acid differences between XJ17-5 and JSTZ1712-12 that might be associated with the distinct virulence. The identification of highly homologous HP-PRRSV variants with new genetic feature and distinct virulence contributes to further analyze the pathogenesis and evolution of PRRSV in the field.

## 1. Introduction

Porcine reproductive and respiratory syndrome (PRRS) has been a critical economic disease in the global swine industry for three decades. The annual cost caused by PRRSV was estimated to be $664 million in national breeding and growing pig herds in the United States alone [1]. PRRSV causes severe reproductive failure in pregnant sows and respiratory distress in neonatal pigs [2]. The etiological agent, PRRS virus (PRRSV), is an enveloped, positive-sense, single-stranded RNA virus classifying within the family *Arteriviridae* [3]. PRRSV genome is ~15kb in length and encodes at least 10 open reading frames (*ORFs*), including *ORF1a*, *ORF1b*, *ORF2a*, *ORF2b*, *ORF5a* and *ORF3-7*. *ORF1a* and *ORF1b* encode at least 16 nonstructural proteins (*nsps*) that are critical for viral replication and transcription, while *ORF2-7* encode eight structural proteins to constitute the viral particle [3,4].

PRRSV is one of the most rapidly evolving RNA viruses [5]. Different PRRSV isolates are antigenically, genetically and pathologically distinct [6]. PRRSV isolates can be divided into PRRSV1 and PRRSV2 species [7]. In Chinese swine herds, PRRSV2 isolates are predominant, while PRRSV1 isolates may also be sporadically detected [8,9]. Three major subtypes of PRRSV2 isolates co-exist in Chinese swine herds including classical PRRSV2, highly pathogenic PRRSV (HP-PRRSV) and NADC30-like PRRSV [10,11,12]. Classical PRRSV2 isolates (CH-1a, S1 and BJ-4) that first emerged in China in 1996 are low virulent [10,13]. HP-PRRSV isolates (JXA1, HuN4 and TJ) first emerged in China in 2006, which are characterized by high fever (40–42 °C), high morbidity (50–100%) and high mortality (20–100%) in all ages of pigs [11,14,15]. HP-PRRSV isolates have a genetic hallmark of discontinuous 30-amino-acid deletion in *nsp2*, but it is not related to the high virulence of HP-PRRSV [16]. NADC30-like PRRSV isolates (JL580, CHsx1401 and HNjz15) first emerged in China in 2013 [12]. Their virulence is quite variable but relatively lower than HP-PRRSV isolates [17]. The unique genetic feature of NADC30-like PRRSV isolates is a discontinuous 131-amino-acid deletion in *nsp2* [12,18,19].

In this study, two novel HP-PRRSV variants were isolated from a diseased pig from Xinjiang province and a healthy pig from Jiangsu province in 2017. Both HP-PRRSV variants have the discontinuous 30-amino-acid deletion and an additional 120-amino-acid deletion in *nsp2*. Genomic sequencing identified that they share high genomic homology. Intriguingly, animal challenge study showed that they have distinct virulence.

## 2. Materials and Methods

### 2.1. Sample Collection and Differential Detection

In April 2017, an outbreak characterized by high fever and severe abortions occurred in a large-scale pig farm with ~4500 sows in Xinjiang province, China. The outbreak caused >1000 abortions and the deaths of >100 sows and >10,000 weaned piglets within half a year. To investigate the causative agent for the outbreak, a total of 17 sera from fever piglets and 9 lungs from diseased and euthanized piglets were frozen and submitted to the Animal Hospital at Yangzhou University. In December 2017, a total of 18 sera were submitted from a clinically healthy pig farm with ~200 sows in Taizhou city, Jiangsu province, China for routine epidemiological investigation. The sera and lungs were used as templates for the routine detection of common swine viruses including PRRSV, classical swine fever virus (CSFV), porcine epidemic diarrhea virus (PEDV), pseudorabies virus (PRV), porcine parvovirus (PPV) and porcine circovirus 2 (PCV2) by conventional and real-time RT-PCR assays [9,20,21,22].

### 2.2. Virus Isolation and Genome Sequencing

To further analyze the novel PRRSV variants identified in this study, a positive lung homogenate from Xinjiang province and a positive serum sample from Jiangsu province were used for virus isolation in Marc-145 cells as previously described [23]. Briefly, the tissue homogenate and the serum sample were inoculated in Marc-145 cells, respectively. The inoculated cells were maintained at 37 °C in a 5% CO_2_ atmosphere and monitored daily for cytopathic effects (CPE). The cultures were frozen at −80 °C when approximately 70% CPE was reached or after 7 days of culturing. The resultant viruses were denominated as XJ17-5 and JSTZ1712-12, respectively. The isolation of PRRSV was confirmed by the indirect immunofluorescence assay (IFA) staining. Marc-145 cells were infected with 200 median tissue culture infectious doses (TCID_50_) of JSTZ1712-12 and XJ17-5, respectively. The infected Marc-145 cells were fixed at 24 h post infection and evaluated by IFA according to the procedure described previously [24]. PRRSV-specific murine mAb 15A1 (1:500 dilution) against the N protein was used as the primary antibody, while the Dylight 594 (Goat anti-mouse IgG, 1:1000, Invitrogen, Carlsbad, CA, USA) was used as the secondary antibody.

Total RNA was extracted from the cell culture using an RNeasy Mini Kit (Qiagen, Hilden, Germany) according to the manufacturer’s instructions. The complete genomes were determined using ten pairs of primers amplifying overlapped fragments as previously reported [23] (Table S1). The amplicons were purified with an E.Z.N.A. Gel Extraction Kit (Omega, Mansfield, TX, USA) and cloned into pEASY-T1 Vector (Transgen, Beijing, China). At least three recombinant clones for each fragment were sequenced by the GENEWIZ Company (Suzhou, China). The obtained sequences were assembled by the DNAMAN 6.0 software and the complete genomes of XJ17-5 and JSTZ1712-12 were deposited in the GenBank database with the accession numbers of MK759853 and MK906026, respectively.

### 2.3. Multiple Alignments, Phylogenetic and Recombination Analyses

To determine the similarity between XJ17-5 and JSTZ1712-12 isolates, the complete genome and each fragment alignments were performed using the DNAMAN 6.0. To analyze the evolutionary relationship between our isolates and other Chinese isolates, a total of 50 representative PRRSV genomes were obtained from the GenBank database and multiplex sequence alignment was generated using Clustal X [25]. Genome-based phylogenetic analysis was performed using MEGA 6.06 [26]. Phylogenetic tree was constructed from aligned genomes using the neighbor-joining method as previously described [27]. The robustness of the phylogenetic tree was evaluated by bootstrapping using 1000 replicates. In addition, the potential recombination events in XJ17-5 and JSTZ1712-12 isolates were screened using the aligned genomes by RDP4 and SimPlot 3.5.1 as previously described [23,28,29]. Briefly, seven methods embedded in RDP4 software, including RDP, GENECONV, BootScran, Maxchi, Chimaera, SiScan and 3Seq were used to detected recombination events and their beginning and ending breakpoints. The default settings were used for all the seven methods, and the highest acceptable *p* value was set at 0.05. In addition, the detected recombination events were further confirmed by SimPlot 3.5.1 using a potential recombinant virus as the query virus and the potential parental viruses as the reference viruses.

### 2.4. Animal Challenge Study

Considering that XJ17-5 and JSTZ1712-12 isolates shared high genomic homology but were isolated from clinically diseased and healthy pigs, respectively, we determined their virulence by animal challenge study. The challenge study was approved (April 7, 2018) by the Animal Welfare and Ethics Committee at College of Veterinary Medicine of Yangzhou University with the reference number of YZU-CVM-201806. Fifteen 4-week-old PRRSV-free piglets were randomly divided into three groups (five piglets per group). Piglets in two groups were intranasally and intramuscularly inoculated with 2 mL 10^5.0^ median tissue culture infectious doses (TCID_50_)/mL XJ17-5 (passage 3) and JSTZ1712-12 (passage 3), respectively, while piglets in the third group were inoculated with Minimum Essential Medium Eagle (MEM media) to serve as the negative control.

Rectal temperature and clinical signs were recorded daily. Serum samples were collected at 0, 4, 7, 11 and 14 days post infection (dpi) for the analyses of virus load and antibody level. The dynamics of viremia were analyzed by real-time RT-PCR [22]. PRRSV-specific antibodies in the sera were detected by HerdCheck^®^ PRRS×3 ELISA Kit (IDEXX, Westbrook, ME, USA). The threshold for seroconversion was set at sample-to-positive (s/p) ratio of 0.4 according to the manufacture’s instruction. The pigs survived until 14 dpi were euthanized and tissue samples were collected for histopathological and immunohistochemical examinations [11,23].

### 2.5. Statistical Analysis

The data of rectal temperature, virus load, antibody level and body weight were shown in means ± standard deviations (SD). The differences between groups were determined by Mann–Whitney *U* Test using Graphpad Prism version 6.07 [23]. A *p* value < 0.05 was considered statistically significant.

## 3. Results

### 3.1. Clinical Sample Detection

Twelve out of 17 sera and 6 out of 9 lungs from Xinjiang province were detected as PRRSV positive, and 2 out of 18 sera from Jiangsu province were detected as PRRSV positive, while all the other pathogens were not detected. *ORF5* sequencing showed that PRRSV from all Xinjiang positive samples shared 100% nucleotide identity and the two Jiangsu positive samples also shared 100% nucleotide identity. Remarkably, *ORF5* sequences from the positive samples from Xinjiang and Jiangsu provinces shared 99.83% nucleotide identity. In addition, *nsp2* sequencing showed that their *nsp2* shared 99.33% nucleotide identity. The high similarities in both PRRSV most variable genes (*ORF5* and *nsp2*) suggested that the viruses from clinically diseased and healthy pig farms are highly homologous.

### 3.2. Virus Isolation and Growth Curve

**Figure 1:**
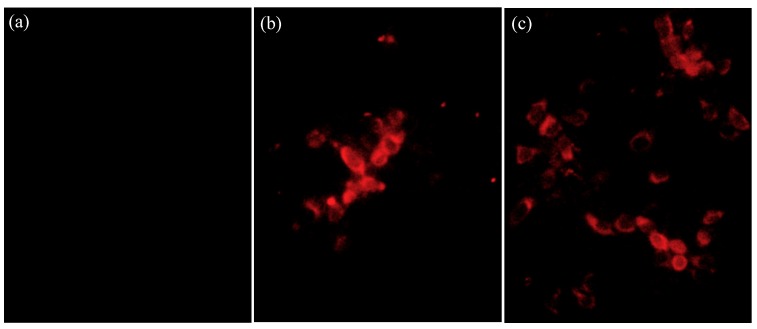
Identification of XJ17-5 and JSTZ1712-12 isolates in Marc-145 cells by the immunofluorescence assay (IFA) staining. Marc-145 cells were infected with 200 median tissue culture infectious doses (TCID_50_) of XJ17-5 and JSTZ1712-12, respectively. The infected Marc-145 cells were fixed at 24 h post infection and evaluated by IFA according to the standard procedure [24]. Porcine reproductive and respiratory syndrome virus (PRRSV)-specific murine mAb 15A1 (1:500 dilution) against the N protein was used as the primary antibody, while the Dylight 594 (Goat anti-mouse IgG, 1:1000, Invitrogen) was used as the secondary antibody. PRRSV-specific antigen could not be detected in mock infected (**a**) Marc-145 cells but could be detected in XJ17-5-infected (**b**) and JSTZ1712-12-infected (**c**) Marc-145 cells. Original magnification at 200×.

**Figure 2:**
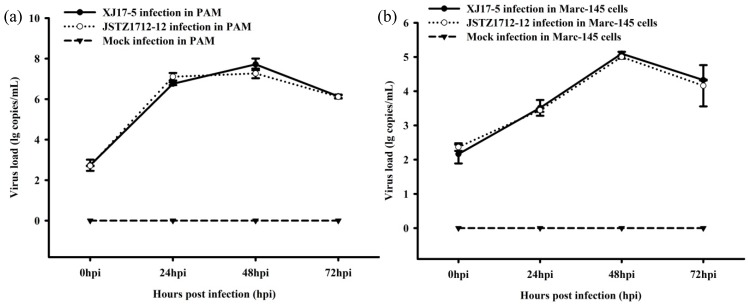
The in vitro replication of JSTZ1712-12 and XJ17-5 isolates. The one-step growth curves in pulmonary alveolar macrophages (PAM) (**a**) and Marc-145 cells (**b**) within 72 h post infection (hpi) were determined by real-time RT-PCR assay [22]. No significant difference was detected in in vitro replication of JSTZ1712-12 and XJ17-5 isolates.

The XJ17-5 and JSTZ1712-12 viruses were successfully isolated in Marc-145 cells. Typical PRRS-specific CPE could be observed at ~4 dpi. The presence of PRRSV was confirmed by the IFA staining. PRRSV-specific fluorescence could be observed at 24 h post infection in both XJ17-5 and JSTZ1712-12 infected Marc-145 cells but not in mock infected Marc-145 cells (Figure 1). In addition, one-step growth curves of XJ17-5 and JSTZ1712-12 in pulmonary alveolar macrophages (PAM) and Marc-145 cells were also determined, which showed that the replication efficacies of XJ17-5 and JSTZ1712-12 in vitro are not significantly different (*p* > 0.05) (Figure 2). The area under the curves (AUC) for virus load versus time were also calculated as previously described [30,31]. The AUC values between XJ17-5 and JSTZ1712-12 infections in either PAM or Marc-145 cells are similar (*p* > 0.05) (Table S2), which further supported that XJ17-5 and JSTZ1712-12 strains have similar replication efficacies.

### 3.3. Genomic Comparison

**Figure 3:**
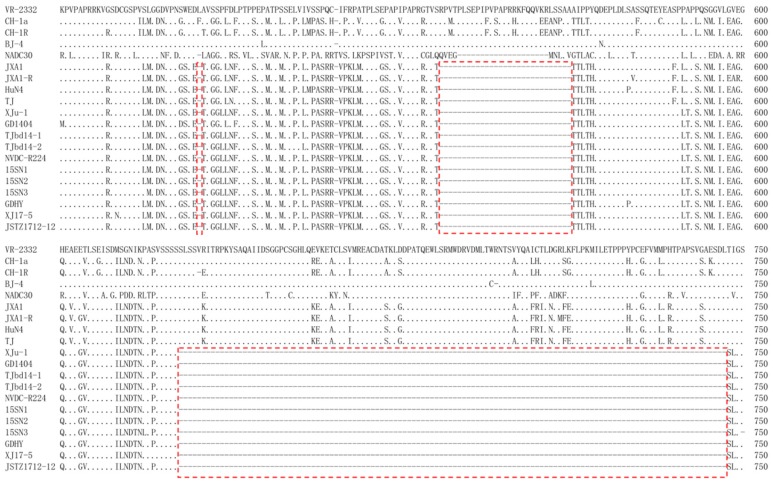
Alignment of *nsp2* amino acid sequences to show the 30-amino-acid discontinuous deletion and the 120-amino-acid continuous deletion. The new genetic feature of 150-amino-acid deletion in XJ17-5 and JSTZ1712-12 isolates and other 9 HP-PRRSV variants are shown in red dashed boxes.

The complete genomes of XJ17-5 and JSTZ1712-12 isolates were determined, which are both 14,960 bp excluding poly (A) tail. XJ17-5 and JSTZ1712-12 genomes share 99.45% nucleotide identity. Genomic comparison with other representative PRRSV strains showed that XJ17-5 and JSTZ1712-12 share 58.31%/58.31%, 86.27%/86.45%, 82.13%/81.93% and 96.46%/96.84% nucleotide identities with representative strains of PRRSV1 (Lelystad virus, LV, M96262), classical PRRSV2 (ATCC VR-2332, PRU87392), NADC30-like PRRSV (NADC30, JN654459) and HP-PRRSV (TJ, EU860248), respectively (Table 1). Each fragment comparison also showed that XJ17-5 and JSTZ1712-12 isolates share the highest nucleotide identity with the HP-PRRSV strain. Both XJ17-5 and JSTZ1712-12 isolates were not detected as recombinant viruses by either RDP4 or Simplot 3.5.1 [23,28,29]. In addition, each fragment alignment identified that both XJ17-5 and JSTZ1712-12 isolates have the discontinuous 30-amino-acid deletion at 481 and 533–561 positions of *nsp2*, which is the genetic hallmark of HP-PRRSV. Remarkably, they also have a continuous 120 amino-acid deletion at 628–747 positions of *nsp2* (Figure 3). The results indicated that XJ17-5 and JSTZ1712-12 isolates are novel HP-PRRSV variants.

### 3.4. Phylogenetic Analysis

**Figure 4:**
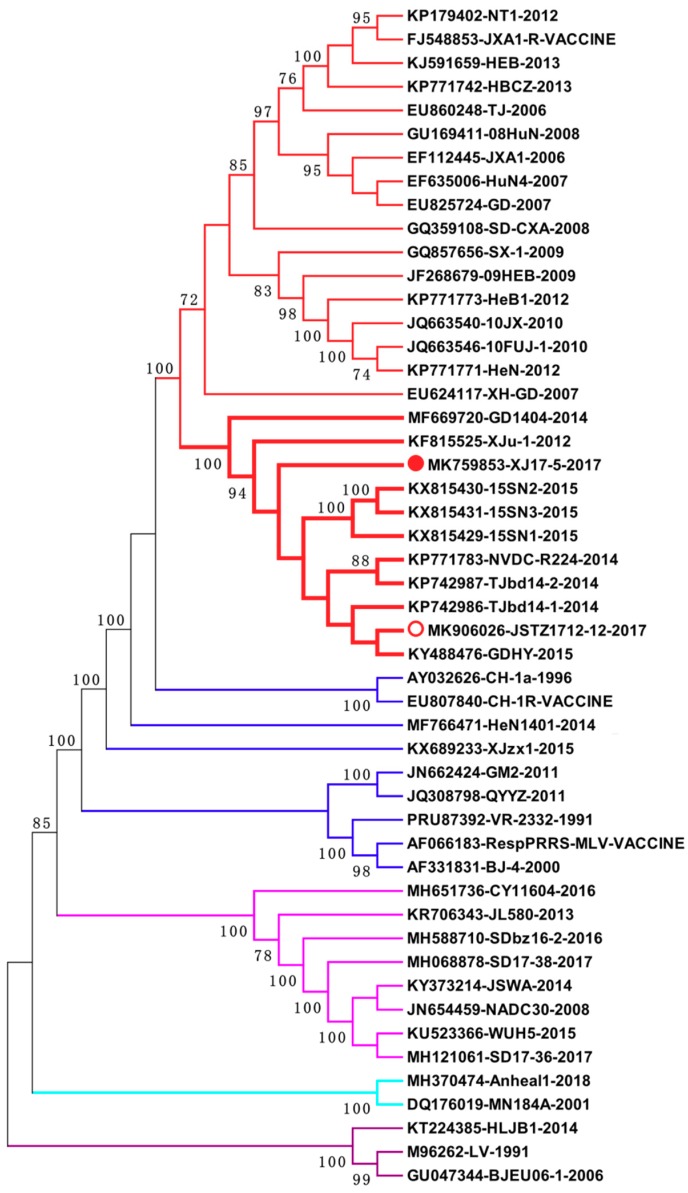
Genome-based genotyping based on 50 representative PRRSV genomes using MEGA 6.06. Our XJ17-5 and JSTZ1712-12 isolates are clustered within the HP-PRRSV subtype and form a new branch with another nine HP-PRRSV variants containing the same 150-amino-acid deletion in *nsp2*. Different types/subtypes are shown in different colors. XJ17-5 and JSTZ1712-12 are highlighted with solid and empty red circles, respectively. The new branch formed by XJ17-5, JSTZ1712-12 and another nine HP-PRRSV variants containing the same 150-amino-acid deletion in *nsp2* is shown in bold. Each virus is presented by the Genbank accession number, the virus name and the year of isolation. Bootstrap values from 1000 replications are indicated for each node, while bootstrap values <70 were not shown.

Fifty-genome-based phylogenetic analysis further supported that XJ17-5 and JSTZ1712-12 are clustered within the HP-PRRSV subtype. However, they formed a new branch with another nine Chinese HP-PRRSV variants (Figure 4). Remarkably, these 11 HP-PRRSV variants all have the same 30-amino-acid discontinuous deletion and 120-amino-acid continuous deletion within *nsp2* (Figure 3). These variants were isolated from five provinces/cities (Xinjiang, Guangdong, Shaanxi, Jiangsu, Tianjin) of China in 2012–2017, suggesting that this new group of HP-PRRSV variants has been circulating in Chinese swine herds for several years.

### 3.5. Pathogenic Analysis

**Figure 5:**
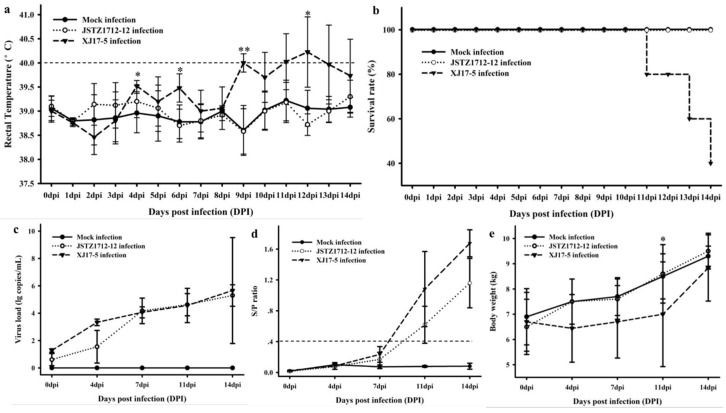
Dynamics of rectal temperature, survival rate, virus load, antibody level and body weight during the challenge study. XJ17-5-infected pigs showed high fever (all pigs ≥40 °C with the highest being 41.2 °C), while JSTZ1712-12-infected and mock-infected pigs were all lower than 40 °C. The significantly higher rectal temperature in XJ17-5-infected pigs was shown with the asterisk (*: *p* < 0.05, **: *p* < 0.01) (**a**). XJ17-5 infection resulted in 60% mortality within 14 dpi, while JSTZ1712-12-infected and mock-infected pigs all survived during the challenge study (**b**). Viremia was analyzed using the real-time RT-PCR assay [22]. The virus could be detected in all XJ17-5-infected pigs and JSTZ1712-12-infected pigs from 4 dpi and 7 dpi, but not in mock-infected pigs (**c**). PRRSV-specific antibody was detected by IDEXX HerdCheck^®^ PRRS×3 Antibody Detection ELISA kit. The threshold for seroconversion was set at sample-to-positive (s/p) ratio of 0.4. PRRSV-specific antibody could be detected in all XJ17-5-infected and JSTZ1712-12-infected pigs from 11 dpi (**d**). XJ17-5-infected pigs have significantly lower body weight than the mock-infected pigs at 11 dpi (*p* < 0.05), while JSTZ1712-12-infected pigs have no significantly difference in body weight comparing with the mock-infected pigs (**e**).

Clinical signs including dyspnea, anorexia and diarrhea were observed in XJ17-5-infected pigs but not in JSTZ1712-12-infected or mock-infected pigs. The rectal temperature of JSTZ1712-12-infected and mock-infected pigs was always lower than 40 °C during the period of animal challenge study; however, the rectal temperature of all five XJ17-5-infected pigs reached ≥40 °C (the highest, 41.2 °C) during the challenge study (Figure 5a). Three XJ17-5-infected pigs died at 11 dpi, 13 dpi and 14 dpi, respectively, while all JSTZ1712-12-infected and mock-infected pigs survived healthy until the end of the study (Figure 5b). Viremia and PRRSV-specific antibody could be detected in all XJ17-5-infected and JSTZ1712-12-infected pigs since 7 dpi and 11 dpi, respectively (Figure 5c,d). The XJ17-5-infected pigs had significantly lower body weight than mock-infected pigs at 11 dpi, but there was no significant difference in body weights between JSTZ1712-12-infected pigs and mock-infected pigs (Figure 5e).

**Figure 6:**
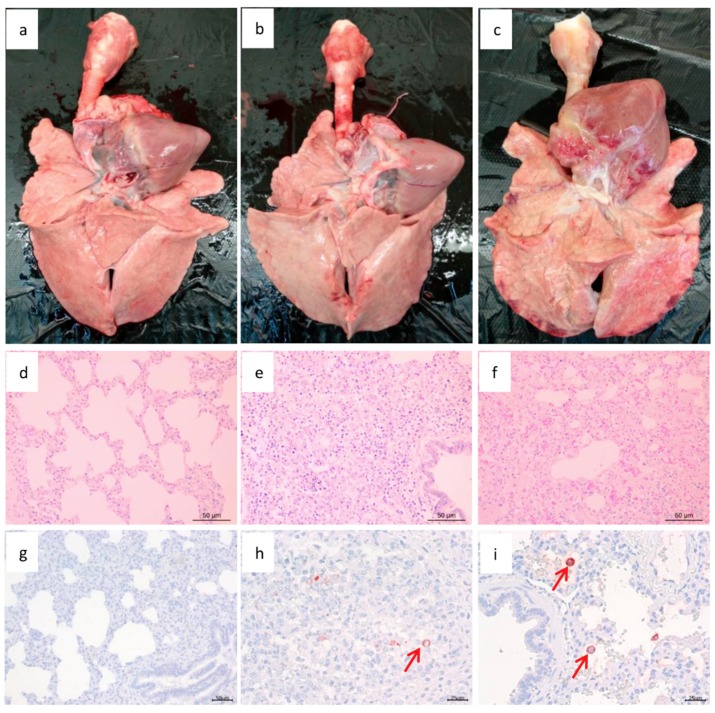
Lung gross lesion, histopathological and immunohistochemical examinations. Lung consolidation and extensive hemorrhages were observed in XJ17-5-infected pigs but not in JSTZ1712-12-infected or mock-infected pigs (**a**–**c**). In the histopathological examination, red blood cells and serous exudation were obviously observed only in XJ17-5-infected pigs (**f**). In addition, interstitial pneumonia with infiltration of lymphocytes and macrophages could also be observed in the lungs of XJ17-5-infected and JSTZ1712-12-infected pigs but not in mock-infected pigs (**d**–**f**). PRRSV antigens could be detected in both JSTZ1712-12-infected and XJ17-5-infected pigs but not in mock-infected pigs in the immunohistochemical examination (**g**–**i**). The red arrows point the positive PRRSV antigen.

During necropsy examination, lung consolidation and extensive hemorrhages were observed in XJ17-5-infected pigs but not in JSTZ1712-12-infected or mock-infected pigs (Figure 6a–c). In the histopathological examination, red blood cells and serous exudation were obviously observed only in XJ17-5-infected pigs (Figure 6f). In addition, interstitial pneumonia with infiltration of lymphocytes and macrophages could also be observed in the lungs of XJ17-5-infected (severe) and JSTZ1712-12-infected (mild) pigs but not in mock-infected pigs (Figure 6d–f). PRRSV antigens could be detected in both JSTZ1712-12-infected and XJ17-5-infected pigs but not in mock-infected pigs in the immunohistochemical examination (Figure 6g–i). The challenge study indicated that XJ17-5 isolate is highly pathogenic but JSTZ1712-12 isolate is nearly not pathogenic for piglets.

### 3.6. Potential Virulence-Associated Amino Acids

To further evaluate the amino acid changes potentially related to the distinct virulence of XJ17-5 and JSTZ1712-12 isolates, the amino acid differences between XJ17-5 and JSTZ1712-12 were analyzed. Totally 34 differences were identified, including 28 differences within nonstructural proteins (*nsp1α*, *nsp1β*, *nsp2*, *nsp9* and *nsp10*) and 6 differences within structural proteins (*GP3*, *GP4*, *GP5* and *N*) (Table 2). Notably, 21 out of the 34 differences were unique for high virulent XJ17-5 isolate and differed from avirulent JSTZ1712-12 isolate and the other 9 highly homologous HP-PRRSV variants available in GenBank database. A majority of these differences (19 out of 21) in the 9 other HP-PRRSV variants are exactly identical to the avirulent JSTZ1712-12 isolates.

## 4. Discussion

HP-PRRS pandemics caused by HP-PRRSV have seriously influenced the development of the Chinese swine industry since 2006 [11,14]. Even though several control strategies, including the commercial modified live HP-PRRS vaccines, have been widely used, HP-PRRS outbreaks in Chinese swine herds are still not effectively controlled due to the continuous emergence of novel HP-PRRSV variants and limited cross-protection of commercial vaccines against heterologous isolates [6,32,33,34]. In this study, we identified two novel HP-PRRSV variants (XJ17-5 and JSTZ1712-12) that have the new genetic feature of 150-amino-acid deletion in *nsp2*. These new HP-PRRSV variants form a new branch within the HP-PRRSV subtype. Even though XJ17-5 and JSTZ1712-12 isolates share high genomic homology, they have distinct pathogenicity for piglets. The amino acid differences between XJ17-5 and JSTZ1712-12 isolates that are potentially associated with the distinct virulence were also determined.

*Nsp2* contains three major domains: a papain-like protease domain (PLP2) at N terminus, a 500- to 700-amino-acid middle hypervariable region and a C-terminal transmembrane domain [35]. Even though *nsp2* is one of the most variable PRRSV regions, the protease domain and transmembrane domain in *nsp2* are much more conserved than the middle hypervariable region. The size of *nsp2* is quite variable due to the natural deletion and insertion in the middle hypervariable region [11,12,36,37]. The discontinuous 30-amino-acid deletion (positions 481 and 532–560) in *nsp2* is the genetic marker of HP-PRRSV isolates; however, this unique deletion in *nsp2* is not related to the high virulence of HP-PRRSV [16]. Notably, our isolates have same additional 120 amino acid deletion at positions 628 to 747 of *nsp2*. BLAST and phylogenetic analyses showed that at least 11 highly homologous viruses with the new genetic feature have been isolated in different regions of China from 2012 to 2017 [38,39,40]. These variants form a new branch within the HP-PRRSV subtype, suggesting that a new group of HP-PRRSV variants is circulating in Chinese swine herds for a long time. However, the virulence of these new variants was not determined yet.

Different PRRSV isolates have distinct pathogenicity. In general, HP-PRRSV isolates have the highest virulence followed by NADC30-like and classical PRRSV2 isolates [11,17,19,41], while current Chinese PRRSV1 isolates are low virulent [8,42]. Retrospective study showed that our XJ17-5 and JSTZ1712-12 isolates were obtained from clinically diseased and healthy pigs, respectively. Whether these highly homologous HP-PRRSV variants with novel deletion in *nsp2* have distinct virulence becomes an interesting question. Notably, a recent study identified that a new isolate JX2014T2, which contains the same 150-amino-acid deletion in *nsp2*, is highly pathogenic to piglets [24]. However, the similarity between the JX2014T2 and our isolates was unable to be determined because the JX2014T2 sequence is not available. Our challenge study showed that XJ17-5 caused high fever, 100% morbidity and 60% mortality, which is similar to the typical HP-PRRSV isolates [11,43]. However, JSTZ1712-12 did not cause fever, any obvious clinical sign or death during the challenge study, suggesting that JSTZ1712-12 is avirulent in piglet. These results showed that even though XJ17-5 and JSTZ1712-12 share high genomic identity, they have significantly distinct virulence. A previous study showed that a mutant with 131-amino-acid deletion (positions 628–759) in *nsp2* of P129 strain is less virulent than the parental virus in pigs [36]. The deletion in the mutant is largely overlapped with the natural 120-amino-acid deletion in our isolates, which raises a hypothesis that the 120-amino-acid deletion in *nsp2* may be related to the virulence. However, the existence of this deletion in both high virulence and avirulence isolates suggests that the deletion is probably not associated with the virulence.

To analyze the virulence determinants of PRRSV isolates, two methods including sequence comparison and reverse genetic manipulation are commonly used [44]. Several studies compared the genomic sequences of high virulence parental virus and the attenuated vaccine strain obtained from in vitro passage in Marc-145 cells to identify the potential mutations associated with the attenuation [15,45,46,47]. The obtained mutations might be related to virulence determinants; however, they might also be associated with the viral adaptation to Marc-145 cells [48,49]. We identified that there are 34 amino acid differences between XJ17-5 and JSTZ1712-12 isolates. These amino acid changes are not related to in vitro adaptation; therefore, they are more likely correlated with virulence determinants. Due to the limited amounts of samples and the low virus loads in the samples, the third passages of XJ17-5 and JSTZ1712-12 isolates rather than the original samples were used for complete genome sequencing and animal challenge study. Previous studies have proved that few passages will not cause significant changes in either the virulence or the genome of PRRSV [15,47].

Plenty of studies have been performed using the reverse genetics to exchange different genes/regions between two PRRSV isolates that are genetically and pathologically distinct [16,50,51]. A concern about this approach is that virulence determinants of PRRSV isolates are likely strain-specific [44,52,53,54], the virulence determinants for one parental virus might be not the same for the other genetically distinct parental virus. The identification of natural HP-PRRSV variants with high genomic similarity but distinct virulence provides ideal viruses to analyze the virulence determinants of these HP-PRRSV variants. A previous study reported that *nsp9* and *nsp10* contribute to the fatal virulence of HP-PRRSV [51]. Furthermore, two recent studies identified mutations in *nsp9* that play crucial roles in the replication and pathogenicity of HP-PRRSV [55,56]. In this study, two amino acid differences in *nsp9* (G_478_A and I_502_T) and four amino acid differences in *nsp10* (K_109_R, I_191_V, S_297_A and A_316_V) were identified between XJ17-5 and JSTZ1712-12 isolates (Table 2). These amino acid sites are not consistent with the virulence-associated mutations identified in previous studies [51,55,56]. Considering that XJ17-5 and JSTZ1712-12 isolates do not have significant difference in in vitro and in vivo replication (Figure 2 and Figure 5c), the amino acid differences in *nsp9* and *nsp10* identified in this study might be not associated with the replication efficiency or the pathogenicity, suggesting that the differences in other regions may also play roles of virulence determinants. For example, *Nsp3-8* and *ORF5* were reported to contain major virulence determinants [50], the mutations in these regions identified in our study might be associated with distinct virulence. Notably, a majority of the mutations (21 out of 34) spread throughout the genome are unique for high virulent XJ17-5 isolate, and 19 out of the 21 mutations are identical in avirulent JSTZ1712-12 and 9 other high homologous HP-PRRSV variants identified by other research groups. Therefore, it is rational to speculate that these mutations have higher probability to associate with the virulence. The infectious clones of XJ17-5 and JSTZ1712-12 isolates have been successfully constructed and rescued in our laboratory. Chimeric and mutant viruses would be constructed to determine the virulence-associated amino acids in the near future.

## 5. Conclusions

In conclusion, this study isolated two HP-PRRSV variants from a severe abortion farm and a clinically healthy farm in China 2017. The two isolates (XJ17-5 and JSTZ1712-12) share 99.45% genomic identity and both contain a 30-amino-acid discontinuous deletion and a 120-amino-acid continuous deletion in *nsp2*. Genome-based phylogenetic analysis showed that they belong to HP-PRRSV subtype but form a new branch with other HP-PRRSV variants containing the same 150-amino-acid deletion in *nsp2*. Pathogenic analysis showed that XJ17-5 is high virulence causing high fever and 60% mortality, while JSTZ1712-12 is avirulent. Fragment comparisons identified 34 amino acid differences between XJ17-5 and JSTZ1712-12 isolates that might be related to distinct virulence. This study identified highly homologous HP-PRRSV variants with distinct virulence, which contributes to further analyze the pathogenesis and evolution of PRRSV in the field.

## Figures and Tables

**Table 1:** Detailed comparison of XJ17-5/JSTZ1712-12 genomes to representative PRRSV strains.

XJ17-5/JSTZ1712-12 *	XJ17-5/JSTZ1712-12 ^#^	VR-2332	NADC30	TJ	LV
		Nucleotide Identity (%) to XJ17-5/JSTZ1712-12			
Complete (14960)	99.45	86.27/86.45	82.13/81.93	96.46/96.84	58.31/58.31
*5’UTR* (1–189)	99.47	91.01/91.53	90.58/91.10	96.30/96.83	51.80/53.15
*ORF1a* (190–7251)	99.35	82.00/82.31	72.55/72.65	93.82/94.25	53.30/53.31
*Nsp1α* (190–729)	99.63	92.04/92.04	89.81/90.00	99.26/99.63	62.11/61.92
*Nsp1β* (730–1338)	98.36	86.21/86.54	80.62/80.79	98.03/99.01	47.76/47.92
*Nsp2* (1339–4479)	99.33	73.88/74.21	67.26/67.30	88.17/88.60	49.44/49.39
*Nsp2N* (1339–3438)	99.14	66.31/66.75	58.31/58.54	83.41/83.98	41.27/41.16
*Nsp2TF* (1339–3438, 3441–3945)	99.31	70.92/71.82	63.97/64.22	86.11/86.59	47.37/47.17
*Nsp3* (4477–5166)	98.99	88.55/89.28	82.03/82.46	97.83/98.55	56.23/56.52
*Nsp4* (5167–5778)	99.67	89.87/89.87	85.29/84.97	99.67/99.67	60.62/60.95
*Nsp5* (5779–6288)	99.61	89.02/89.41	90.20/90.59	99.02/99.41	63.14/63.33
*Nsp6* (6289–6336)	100	93.75/93.75	91.67/91.67	97.92/97.92	70.83/70.83
*Nsp7α* (6337–6783)	99.55	90.38/90.83	84.56/84.56	98.88/99.33	55.36/55.58
*Nsp7β* (6784–7113)	100	87.88/87.88	79.39/79.39	99.39/99.39	48.66/48.66
*Nsp8* (7114–7248)	100	96.30/96.30	89.63/89.63	100/100	64.44/64.44
*ORF1b* (7248–11621)	99.47	91.08/91.06	87.93/87.86	98.88/99.22	62.11/62.14
*Nsp9* (7248–9167)	99.32	92.45/92.29	87.60/87.34	98.54/98.91	66.35/66.35
*Nsp10* (9168–10487)	99.39	89.85/90.00	85.76/85.91	98.79/99.24	60.30/60.30
*Nsp11* (10488–11159)	99.70	90.18/90.18	91.22/91.22	99.26/99.55	65.33/65.63
*Nsp12* (11160–11618)	100	89.39/89.39	89.83/89.83	99.13/99.13	46.15/46.27
*ORF2a* (11623–12393)	99.74	93.13/93.39	86.64/86.38	99.35/99.61	62.13/63.55
*ORF2b* (11628–11849)	100	93.24/93.24	87.67/87.67	100/100	68.72/68.72
*ORF3* (12246–13010)	99.74	89.15/89.41	83.14/83.40	98.95/99.22	61.92/62.17
*ORF4* (12791–13327)	99.63	89.76/90.13	86.78/87.15	99.07/99.44	64.49/64.67
*ORF5* (13338–13940)	99.83	89.05/88.89	86.24/86.07	99.17/99.34	61.33/61.17
*ORF5a* (13328–13468)	100	88.65/88.65	85.82/85.82	98.58/98.58	55.32/55.32
*ORF6* (13925–14449)	99.62	95.43/95.05	89.33/88.95	99.43/99.81	69.14/69.14
*ORF7* (14439–14810)	98.92	93.28/93.55	90.32/90.59	98.92/99.46	62.37/60.86
*3’UTR* (14811–14960)	98.67	92.05/93.38	88.08/89.40	98.67/100	55.63/54.30

* The lengths of the complete genome and each fragment of XJ17-5 isolate (MK759853) and JSTZ1712-12 isolate (MK906026) are identical. ^#^ XJ17-5 and JSTZ1712-12 isolates share the highest nucleotide identity with each other at the complete genome and each fragment rather than any other representative PRRSV strains including VR-2332 (PRU87392), NADC30 (JN654459), TJ (EU860248) and Lelystad virus (LV) (M96262).

**Table 2:** Distinct amino acids between XJ17-5 and JSTZ1712-12 isolates and corresponding sites in 9 other highly homologous highly pathogenic (HP)-PRRSV variants.

No.	Protein	Position *	XJ17-5	JSTZ1712-12	XJu-1	GD1404	NVDC-R224	TJbd14-1	TJbd14-2	GDHY	15SN1	15SN2	15SN3
1	*Nsp1α*	114	A ^#^	T	T	T	T	T	T	T	T	T	T
2	*Nsp1β*	8	C	R	R	R	R	R	R	R	R	R	R
3		83	N	D	D	D	D	D	D	D	D	D	D
4		107	I	F	I	I	I	I	I	I	I	I	I
5		122	A	P	P	A	S	S	S	P	P	P	P
6		137	A	T	T	A	T	A	A	T	A	A	A
7	*Nsp2*	26	V	I	I	T	I	I	I	I	I	I	I
8		147	E	G	E	E	E	E	E	E	E	E	E
9		335	F	S	S	S	S	S	S	S	F	F	F
10		355	V	A	A	V	V	V	V	A	V	V	V
11		363	I	V	V	V	V	V	V	V	V	V	V
12		393	K	E	E	E	E	E	E	E	E	E	G
13		463	N	D	D	D	D	D	D	D	D	D	D
14		590	S	P	P	P	P	P	P	P	P	P	P
15		604	G	D	D	D	D	D	D	D	D	D	-
16		640	S	P	P	P	P	P	P	P	P	P	P
17		999	E	D	E	E	E	D	E	E	E	E	E
18	*Nsp3*	32	T	A	A	A	A	A	A	A	A	A	A
19		102	T	A	A	A	A	A	A	A	A	A	A
20	*Nsp4*	184	K	N	K	N	N	N	N	N	N	N	N
21	*Nsp5*	53	A	V	V	V	V	V	V	V	V	V	V
22	*Nsp7α*	8	I	M	M	M	M	M	M	M	M	M	M
23	*Nsp9*	478	G	A	A	A	A	A	A	A	A	A	A
24		502	I	T	T	I	I	I	I	I	I	I	I
25	*Nsp10*	109	K	R	R	R	R	R	R	R	R	R	R
26		191	I	V	V	V	V	V	V	V	V	V	V
27		297	S	A	A	S	S	A	S	A	S	S	S
28		316	A	V	V	V	V	V	V	V	V	V	V
29	*GP3*	69	S	P	P	P	P	P	P	P	P	P	P
30		228	P	S	S	S	S	S	S	S	S	S	S
31	*GP4*	46	A	V	V	V	V	V	V	V	V	V	V
32	*GP5*	200	P	L	L	L	P	L	P	L	L	L	L
33	*N*	51	E	G	G	E	G	G	G	G	G	G	E
34		123	V	A	A	A	A	A	A	A	A	A	A

* The positions are determined based on each encoded protein of XJ17-5 isolate (MK759853). ^#^ The animo acids that are unique in XJ17-5 and differ from all the other 10 highly homologous HP-PRRSV variants are shown in bold and underlined.

## Supplementary Materials

The following are available online at https://www.mdpi.com/1999-4915/11/9/875/s1, Table S1: Primers used for PRRSV2 genome amplification. Table S2. AUC values for virus load versus time in PAM and Marc-145 cells.
