# Supplementary material for: Identification of Two Porcine Reproductive and Respiratory Syndrome Virus Variants Sharing High Genomic Homology but with Distinct Virulence
Source: Viruses. 2019 Sep 18;11(9):875. doi: 10.3390/v11090875 (PMC6783987; doi:10.3390/v11090875)
Supplement: Supplementary file 1 [file viruses-11-00875-s001.pdf]

Table S1. Primers used for PRRSV2 genome amplification.

Primer	Sequence (5’-3’)	location[a]	Length of amplicon
PRRSV-1F	ATGACGTATAGGTGTTGGCTCTATGC	1-26	1641 bp
PRRSV-1R	AGGGAGCCTGAGGATTTGGAT	1621-1641	
PRRSV-2F	ATCGCCAACCGGATGGT	1522-1538	1747 bp
PRRSV-2R	CGATGATGGCTTGAGCTGAGTA	3247-3268	
PRRSV-3F	TGTCATCAAGCAGCTCCCTGT	3200-3220	1661 bp
PRRSV-3R	AAGGACGAGGTTCGCGGT	4843-4860	
PRRSV-4F	TTTCCCGCTGGAGTGAAAGTT	4756-4776	1688 bp
PRRSV-4R	GCTGTCAGAAGCCTGATCATCAG	6421-6443	
PRRSV-5F	AATGAGATTCTCCCAGCTGTCCT	6331-6353	1757 bp
PRRSV-5R	GCGCCTAATATCACAAGCCTGTAT	8064-8087	
PRRSV-6F	GGAAACACTGGGATTGATGGC	7983-8003	1760 bp
PRRSV-6R	CCACACCAGATTATAACAGGACAATG	9717-9742	
PRRSV-7F	CGTACGCCACTGCCTGTG	9661-9678	1663 bp
PRRSV-7R	GGGAGGGACTCAGCAACTTCT	11303-11323	
PRRSV-8F	TGCTTCCGGAGACAGTCTTCA	11230-11250	1749 bp
PRRSV-8R	GAACCATGAACCCTAGTTCGTCAT	12955-12978	
PRRSV-9F	TGAACTCATGGTGAATTACACGGT	12830-12853	1619 bp
PRRSV-9R	GTAATGGAAAACGCCAAAAGCA	14427-14448	
PRRSV-10F	GAGTTGTGCTTGATGGTTCCG	14320-14340	1093 bp
PRRSV-10R	TAATTACGGCCGCATGGTTC	15393-15412	

[a] The location is determined based on the representative strain ATCC VR-2332 (PRU87392).

Table S2. AUC values for virus load versus time in PAM and Marc-145 cells.

Group	In PAM			In Marc-145 cells		
	Mean AUC (minimum, maximum)	*p*-value (vs mock infection)	*p*-value (vs XJ17-5 infection)	Mean AUC (minimum, maximum)	*p*-value (vs mock infection)	*p*-value (vs XJ17-5 infection)
Mock infection	0 (0,0)	N/A	<0.01	0 (0,0)	N/A	<0.01
XJ17-5 infection	18.91 (19.44, 18.38)	<0.01	N/A	11.86 (12.29, 11,43)	<0.01	N/A
JSTZ1712-12 infection	18.78 (19.25, 18.31)	<0.01	0.41	11.71 (12.17, 11,25)	<0.01	0.38
